# Biopsy samples from patients with irritable bowel syndrome, but not from those with mastocytosis or unspecific gastrointestinal complaints reveal unique nerve activation in all gut regions independent of mast cell density, histamine content or specific gastrointestinal symptoms

**DOI:** 10.3389/fnins.2024.1291554

**Published:** 2024-07-02

**Authors:** Sheila Vignali, Sabine Buhner, Wolfgang Greiter, Hannelore Daniel, Thomas Frieling, Michael Schemann, Anita Annahazi

**Affiliations:** ^1^Chair of Human Biology, Technical University of Munich, Freising, Germany; ^2^Chair of Zoology, Technical University of Munich, Freising, Germany; ^3^Chair of Nutrition Physiology, Technical University of Munich, Freising, Germany; ^4^Medical Clinic II, Helios Klinikum Krefeld, Krefeld, Germany

**Keywords:** irritable bowel syndrome, mastocytosis, enteric neurons, mast cells, neuroimaging, small bowel

## Abstract

**Introduction:**

We previously showed enteric nerve activation after application of colonic mucosal biopsy supernatants from patients with irritable bowel syndrome (IBS). The question remains whether this is a region-specific or a generalized sensitization. We tested the nerve-activating properties of supernatants from large and small intestinal regions of IBS patients with diarrhea (IBS-D) in comparison to those from mastocytosis patients with diarrhea (MC-D) or non-IBS/non-MC patients with GI-complaints. MC-D patients were included to test samples from patients with an established, severe mast cell disorder, because mast cells are suggested to play a role in IBS.

**Methods:**

Voltage-sensitive dye imaging was used to record the effects of mucosal biopsy supernatants from IBS-D, MC-D, and non-IBS/non-MC on guinea pig submucous neurons. Mast cell density and histamine concentrations were measured in all samples.

**Results:**

The median neuroindex (spike frequency × % responding neurons in Hz × %) was significantly (all *p* < 0.001) increased for IBS-D (duodenum and colon, proximal and distal each, 49.3; 50.5; 63.7; 71.9, respectively) compared to non-IBS/non-MC (duodenum and colon, proximal and distal each, 8.7; 4.9; 6.9; 5.4, respectively) or MC-D supernatants (duodenum and colon, proximal and distal each, 9.4; 11.9; 0.0; 7.9, respectively). Nerve activation by MC-D and non-IBS/non-MC supernatants was comparable (*p*>0.05). Mast cell density or histamine concentrations were not different between IBS-D, MC-D, and non-IBS/non-MC samples.

**Discussion:**

Nerve activation by biopsy supernatants is an IBS hallmark that occurs throughout the gut, unrelated to mast cell density or histamine concentration. At least as important is our finding that GI complaints *per se* were not associated with biopsy supernatant-induced nerve activation, which further stresses the relevance of altered nerve behavior in IBS.

## 1 Introduction

Irritable bowel syndrome (IBS) is a disorder of gut-brain interaction, characterized by abdominal pain, bloating, and altered bowel habits with constipation, diarrhea, or both (Drossman, 2016). The definition and diagnosis of IBS are based on the ROME criteria (Drossman, 2016). The pathogenesis of IBS is complex and still not completely understood. It involves central and peripheral factors, such as disturbed motility, visceral hypersensitivity and increased cortical pain perception, impaired gut barrier function, microbiota changes, and low-grade gut wall inflammation (Enck et al., 2016). One important element is the interaction between neurons and immune cells within the intestinal wall. It has been shown that soluble factors released from colonic mucosal biopsy specimens of IBS patients activate visceral afferent neurons in rats and mice and induce somatic and visceral hypersensitivity (Barbara et al., 2007; Cenac et al., 2007). We previously demonstrated activation of enteric neurons in both the myenteric and submucous neurons after the application of colonic mucosal biopsy supernatants from IBS patients (Buhner et al., 2009, 2012). The most important components of the supernatants responsible for this effect are proteases, signaling *via* protease-activated receptor (PAR)-1 on human submucous enteric neurons (Buhner et al., 2018). The effect of two further components, serotonin and histamine is less pronounced, but tryptase potentiates their nerve-activating properties, providing an explanation of how a mixture of low amounts of secreted mediators could affect neuronal functions *in vivo* in the colonic wall (Ostertag et al., 2017). Although studies usually focus on the colon, possible involvement of small bowel in IBS is suggested as symptoms are often associated with food, and lymphocytic infiltration has been described in different segments of the small bowel in IBS patients (Burns et al., 2022). Recently, disintegration of the mucosa and recruitment of intraepithelial lymphocytes have been shown as an immediate response to topical application of food allergens in the duodenum in a proportion of IBS patients (Fritscher-Ravens et al., 2014). In addition, duodenal epithelial barrier dysfunction has been shown in IBS (Frieling et al., 2022). Furthermore, lipid infusion into the duodenum evoked significantly more abdominal symptoms in IBS patients compared to controls, and duodenal TRPV1 expression correlated with abdominal pain and rectal hypersensitivity in these patients (Grover et al., 2021). The overlap between functional dyspepsia and IBS can be as high as 55%, suggesting a disturbance of gut-brain interaction throughout the gastrointestinal tract (Barberio et al., 2022). However, it is unclear whether the phenomenon of visceral hypersensitivity is a general feature throughout the gut or restricted to the colon.

Different studies indicate that the symptoms of IBS are associated with an increased mucosal mast cell number and increased release of their mediators (Barbara et al., 2004; Buhner et al., 2009; Frieling et al., 2011). A mediator cocktail released from activated mast cells stimulated guinea pig and human enteric neurons (Schemann et al., 2005). Furthermore, the release of mast cell mediators such as histamine and proteases were increased in IBS patients (Barbara et al., 2004; Frieling et al., 2011) and led to an activation of sensory (Barbara et al., 2007) and enteric neurons (Buhner et al., 2009, 2012). However, other studies found no change (Spiller, 2000; Kerckhoffs et al., 2012; El-Salhy et al., 2013) or even a decrease in the number of mast cells in the mucosa of IBS patients (Braak et al., 2012) (for a review see Schemann and Camilleri, 2013). The role of mast cells in the symptom generation in IBS patients remains also unclear, as some studies found a positive correlation between the number of mast cells and clinical parameters (Barbara et al., 2004; Piche et al., 2007; Martínez et al., 2013), but others did not (O'Sullivan et al., 2000; Park et al., 2006; Bednarska et al., 2017). Interestingly, serotonin content of jejunal biopsies from IBS-C patients were significantly lower, while the number of mucosal mast cells in both IBS-D and IBS-C patients was significantly higher in the ileum compared to healthy controls, supporting a possible involvement of the small bowel in the pathogenesis of the disease (Wang et al., 2007). Additionally, in pediatric IBS patients, the mast cell number was significantly elevated also in the stomach, duodenum, terminal ileum, besides the descending colon (Rizvi et al., 2022). In a cohort of adult IBS patients, mast cell counts were significantly higher in the distal part of the duodenum than in controls, and vitamin D receptor protein expression was also elevated (Miura et al., 2021). However, a previous meta-analysis found increased mast cell counts in the ileum, but not in the duodenum and jejunum of IBS patients (Robles et al., 2019).

Mastocytosis (MC) is a hematologic disorder characterized by abnormal clonal expansion of genetically altered mast cells and their accumulation in different organs (Valent et al., 2017). Currently, mastocytosis is divided in cutaneous mastocytosis (CM), which has no systemic involvement, systemic mastocytosis (SM), and mast cell sarcoma (Leguit et al., 2022). SM is diagnosed based on the presence of the major WHO criterion or, in its absence, by the presence of at least three minor WHO criteria (Leguit et al., 2022). If mastocytosis is coupled with mast cell activation, the excess release of mast cell mediators results in diverse symptoms affecting various organ systems. In the gastrointestinal tract for example this leads to an increased fluid secretion into the intestinal lumen, increased peristalsis and thus to diarrhea and vomiting (Molderings et al., 2005; Weinstock et al., 2021). In our study, we selected MC patients who suffered from abdominal symptoms similar to the patients in the IBS group, i.e., diarrhea and abdominal pain (MC-D).

We aimed to answer two questions in our study. First, to explore if mucosal biopsy supernatants from small and large intestinal regions of IBS-D patients similarly activate enteric nerves. Second, to compare mast cell density, concentration of histamine in supernatants as well as their nerve-activating properties in IBS-D, MC-D, and non-IBS/non-MC patients, who also suffered from gastrointestinal symptoms.

## 2 Materials and methods

### 2.1 Biopsy samples

Patients (*n* = 32; 6 MC, 16 IBS-D, 10 non-IBS/non-MC) were recruited in the Helios Klinikum Krefeld. The protocols and procedures performed on human subjects and samples were approved by the Ethics Committee of Heinrich-Heine-Universität Düsseldorf under study no. 3166 and conformed to the standards set by the Declaration of Helsinki and its later amendments. All persons gave their informed consent prior to their inclusion in the study. Mucosal biopsy samples were taken from four different intestinal regions for the preparation of supernatants (from the pars descendes duodeni, taken oral and aboral from the papilla of Vater, referred to as proximal and distal duodenum, respectively; from the ascending colon, referred to as proximal colon; and from the sigma, referred to as distal colon) when the patients were symptomatic. Further biopsies were taken from the same regions and from the rectum to assess the mast cell density. IBS-D patients (mean age: 45 ± 4 years) were diagnosed according to the German S3 guideline (Layer et al., 2011) and the ROME II criteria. MC-D patients (mean age: 51 ± 5 years) were diagnosed according to WHO criteria (Horny et al., 2007). Biopsies from the same four intestinal regions were obtained during endoscopies from patients who suffered from gastrointestinal symptoms but were non-IBS/non-MC patients (mean age 65 ± 5 years). It was not possible to perform a biopsy sampling from several regions from healthy volunteers. IBS mostly affects younger people. Thus, patients in the non-IBS/non-MC group were significantly older than IBS-D (*p* < 0.05), but their age was comparable to MC-D patients (*p* > 0.05). There was no difference in age between MC-D and IBS-D patients (*p* > 0.05). If only female patients are included in the analysis, there is no significant difference between the three groups in age [non-IBS/non-MC: 55.0 (48.0/78.0) vs. MC-D: 48.5 (43.0/61.5) vs. IBS-D: 42.0 (37.8/60.0); *p* = 0.295]. In Table 1 all patient data are summarized. Some IBS samples were used previously in another publication to study the effects of serpin (see Table 1) (Buhner et al., 2018).

**Table 1:** Patient information.

**Supernatant #**	**Symptoms**	**Diagnosis**	**Gender**	**Age**
MC 1	Diarrhea, abdominal pain	Cutaneous mastocytosis	♀	51
MC 4	Diarrhea	Cutaneous mastocytosis	♀	59
MC 12	Diarrhea	Cutaneous mastocytosis	♀	69
MC 13	Diarrhea, abdominal pain	Cutaneous mastocytosis	♀	46
MC 23	Diarrhea	Systemic mastocytosis	♀	37
MC 25	Diarrhea, abdominal pain	Cutaneous mastocytosis	♀	45
IBS 5^*^	Diarrhea, abdominal pain	IBS-D	♀	38
IBS 6	Diarrhea, abdominal pain	IBS-D	♀	83
IBS 7^*^	Diarrhea, abdominal pain, bloating	IBS-D	♂	28
IBS 9^*^	Diarrhea, abdominal pain, bloating	IBS-D	♂	50
IBS 10	Diarrhea, abdominal pain, bloating	IBS-D	♀	40
IBS 11^*^	Diarrhea, abdominal pain, bloating	IBS-D	♀	44
IBS 14	Diarrhea, abdominal pain	IBS-D	♀	38
IBS 15^*^	Diarrhea, abdominal pain	IBS-D	♀	53
IBS 17^*^	Diarrhea, abdominal pain	IBS-D	♀	37
IBS 18^*^	Diarrhea, abdominal pain	IBS-D	♀	69
IBS 19	Diarrhea, abdominal pain	IBS-D	♂	25
IBS 21^*^	Diarrhea, abdominal pain, bloating	IBS-D	♂	31
IBS 6	Diarrhea, abdominal pain	IBS-D	♀	32
IBS 27^*^	Diarrhea, abdominal pain	IBS-D	♀	57
IBS 2.2	Diarrhea, abdominal pain	IBS-D	♂	44
IBS 3.2	Diarrhea, abdominal pain	IBS-D	♂	44
Non-IBS/non-MC 2	Heartburn	GERD	♂	67
Non-IBS/non-MC 3	Anemia	Gastrointestinal bleeding	♂	73
Non-IBS/non-MC 4	Bloating	Obstructive defecation syndrome	♀	69
Non-IBS/non-MC 5	Bloating, alternating stool pattern	Functional abdominal bloating	♀	44
Non-IBS/non-MC 6	Bloating, incomplete evacuation	Obstructive defecation syndrome	♂	84
Non-IBS/non-MC 7	Blood in stool	Gastrointestinal bleeding	♀	87
Non-IBS/non-MC 8	Epigastric pain	GERD	♀	55
Non-IBS/non-MC 9	Abdominal pain	Functional abdominal pain	♂	49
Non-IBS/non-MC 16	Heartburn	GERD, food intolerance	♂	66
Non-IBS/non-MC 5.2	Abdominal pain, meteorism	Sorbitol intolerance	♀	52

^*^Samples have also been used in a previous study to test the effect of serpin (Buhner et al., 2018).

Biopsy supernatants were extracted as described previously (Buhner et al., 2018). Briefly, after being removed from the patients, the biopsies were transferred into fresh and sterile reaction tubes filled with 1 ml HEPES/Krebs buffer and incubated at 37°C for 25 min. The supernatants were collected and stored at −80°C. Four biopsies per patient and region were used to collect the supernatants. In contrast, histology was performed in four biopsies from the descending duodenum (aboral of the papilla Vater), four from the ascending colon, four from the sigma region, and four from the rectum.

### 2.2 Neuroimaging of supernatant effects on guinea-pig enteric neurons

For the experiments, we used male Dunkin Hartley guinea pigs (Harlan Laboratories B.V., AN Venray, Netherlands). After euthanizing the animals with a blow to the head followed by exsanguination, the proximal part of the distal colon was quickly removed and dissected in ice-cold Krebs solution by removing the mucosa and the muscular layers to obtain submucous plexus preparations. All animal work was conducted according to the German guidelines for animal care and welfare (Deutsches Tierschutzgesetz) and approved by the Bavarian state Ethics Committee (Regierung Oberbayern, which serves as the Institutional Care and Use Committee for the Technical University of Munich) according to §4 and §11 German Animal Protection Law under reference number 32-568-2. During the preparation the tissue was constantly perfused with ice-cold oxygenated (5% CO_2_, 95% O_2_) Krebs solution (pH 7.4) containing in mmol/L: 117 NaCl, 4.7 KCl, 1.2 MgCl_2_, 1.2 NaH_2_PO_4_, 25 NaHCO_3_, 2.5 CaCl_2_ and 11 glucose. The final preparation 10 × 20 mm) was placed in a recording chamber and continuously perfused with oxygenated 37°C Krebs solution (pH 7.4).

The multi-site optical recording technique is an ultra-fast imaging technique that allows us to record action potential discharge of low and high frequencies in the guinea pig submucous plexus. The details of this technique have been described previously (Neunlist et al., 1999; Schemann et al., 2002; Michel et al., 2005). The freshly dissected tissues were placed in a recording chamber with a 42 mm diameter glass bottom (130–170 μm thickness, Sauer, Reutlingen, Germany) and mounted onto an epifluorescence Olympus IX 71 microscope (Olympus, Hamburg, Germany) equipped with a 75 W xenon arc lamp (Optosource, Cairn Research Ltd., Faversham, UK). Controlled illumination of the preparation for 2 s was achieved by a software-operated shutter (UniblitzD122, Vincent Associates, New York, USA). Individual ganglia were stained with the fluorescent voltage-sensitive dye (VSD) 1-(3-sulfonatopropyl)-4-[β[2-(di-n-octylamino)-6 naphtyl] vinyl] pyridinium betaine (Di-8-ANEPPS; Molecular Probes Mobitec, Göttingen, Germany) by local pressure application through a fine tipped glass pipette loaded with 20 μmol/L Di-8-ANEPPS. Recordings from single neurons were performed with a 100x oil immersion objective (UAPO/340, NA = 1.4; Olympus, Hamburg, Germany) using a fluorescence filter cube consisting of a 545 ± 15 nm excitation interference filter, a 565 nm dichroic mirror, and a 580 nm barrier filter (AHF Analysentechnik, Tübingen, Germany). Signals were acquired with a frequency of 1.6 kHz, which enables the detection of action potentials, and were processed by a cooled charge-coupled device camera made of 80 × 80 pixels (RedShirt Imaging, Decatur, GA, USA). The VSD imaging setup allows measurements of relative changes in the fluorescence (ΔF/F, the denominator F indicates resting light level while ΔF is the relative change in fluorescence) which is linearly related to changes in the membrane potential (Neunlist et al., 1999). The Neuroplex software enabled signal filtering. A high-pass Butterworth filter was used to reduce mainly dye bleaching and some minor mechanical noise. A low-pass Butterworth filter was used to reduce high-frequency electrical noise. The number of action potentials was not affected by filtering.

Proximal colonic, sigmoid and proximal and distal duodenal biopsy supernatants from MC-D, IBS-D patients and non-IBS/non-MC were applied onto single submucous ganglia by pressure ejection from micropipettes (20 psi, 400–600 ms duration, 100–200 μm distances from the ganglion). Before the application of the supernatants, the viability of the neurons within a ganglion was tested by evoking a fast excitatory postsynaptic potential with an electrical stimulus to an interganglionic fiber tract or by recording spontaneous neuronal activity. The experiments were performed at least in three guinea pig tissues, eight ganglia, and 80 neurons for each supernatant and always in the same way. To ensure that the spike discharge was not caused by spontaneous activity, a recording of basal activity was performed before the application of supernatants. The application of samples from IBS-D and MC-D patients was always paired with samples from non-IBS/non-MC from the same intestinal region.

### 2.3 Mast cell counts

The extracted mucosal biopsies were immediately fixed in a 4% formalin solution (buffered neutral) for at least 4 h at room temperature. Subsequently, the fixed tissue samples were dehydrated with an ascending ethanol series and embedded in paraffin after intermediate treatment with xylol. An automated embedding system was used (Tissue-Tek^®^ VIP^®^ 6 Vacuum Infiltration Processor or Tissue-Tek^®^ TEC^®^ 5 Tissue Embedding Console System). The tissue slices were prepared with a sliding microtome with an average thickness of 2–4 μm. The cutting plane of each cut was performed amounting to the mucosa in the lamina propria and the crypts. Subsequently, the sections were transferred to immune staining. The tissue sections were placed in an automated immune staining system (BenchMark XT automated slide preparation system), where each slice was automatically preprocessed and stained. The tissues were first incubated with the primary antibodies for tryptase from the mouse and for the pathway c-Kit from the rabbit over a period of 60 min at room temperature and then incubated with the detection medium, containing the secondary enzyme-labeled antibodies and chromogen for the red color reaction. In order to localize also the nuclei of mast cell surrounding cells, the tissues were stained with hematoxylin. This substance contains an acidic color complex that binds selectively to nucleic acids and histones in the cells resulting in a blue staining of cell nuclei. Finally, the sections were dehydrated gradually with an ascending alcohol series and then covered. To determine the number of mast cells in the stained tissue sections, each section was analyzed at 108-fold magnification using a stereo microscope [Olympus SZX12, Olympus Deutschland GmbH (Hamburg, Germany)] and photographed with a digital camera [Olympus CAMEDIA Digital Camera C-4040 Zoom, Olympus Deutschland GmbH (Hamburg, Germany)]. For a direct comparison between the two stainings (tryptase and c-Kit), always the same regions within the tissue section were reconsidered. The analysis of the resulting images was performed by the image processing program Olympus DP-Soft. The ascertained mast cell number in both stainings was converted with the help of the determined area of counted mast cells to mast cells per square millimeter (mm^2^) using the following equation: mast cell density = number of counted mast cells/area of counted mast cell (1/mm^2^). All values calculated with the help of this equation were tabulated according to bowel region and staining and pooled according to the patient's diagnoses.

### 2.4 Determination of the concentration of histamine

The concentrations of histamine in each supernatant were quantified by a combined flow injection (FIA) and LC-MS/MS assay with a triple-quad mass spectrometer (AB SCIEX QTRAP 5500). For the determination, the AbsoluteIDQ^TM^ p180 Kit from Biocrates in combination with a 100 mm chromatography column (Agilent Zorbax Eclipse XDB C18, 3.0 × 100 mm, 3.5 μm) was used. The AbsoluteIDQ kit was prepared as described in detail in the user manual. Ten microliter of each sample was added to the center of the filter. After 10 min drying in a nitrogen evaporator, 10 μl of a 4-time dilution of the internal standard (ISTD) from Biocrates, which contains deuterium-labeled standards for histamine, was added to the center of the filter and dried again in a nitrogen evaporator. Subsequently, 40 μl of a 5% solution of phenylisothiocyanate (PITC), containing liquid chromatography mass spectrometry (LCMS) H_2_O, ethanol, and pyridine, was added for the derivatization to each dried sample. After incubation at room temperature for 20 min and being shaken at 450 rpm, the filter spots were dried again for at least 45 min using a nitrogen evaporator. The samples were then extracted in 140 μl of a 5 mM ammonium acetate solution in methanol and incubated at room temperature and 450 rpm for 30 min. The single extract was obtained by centrifugation into fresh caps. For the spectrometric analysis, each sample was diluted with LCMS H_2_O to obtain a dilution relation of 7 (for the sample solution) and 3 (H_2_O) before being applied to the column.

Since present samples can contain histamine concentrations different from those in standard human biosamples (1–80 μM) a new calibration was constructed with deuterium-labeled d_4_-histamine (Histamine-α, α, β, β-d_4_ 2HCl) as internal standard and the following histamine concentration: 0; 0.05; 0.1; 0.25; 0.5; 1.0 μM. To simulate the same matrix condition as in the biopsy supernatants, we created a so-called bulk sample, a mixture of many different biopsy samples as background. For the spectrometric analysis, the column was automatically perfused with H_2_O/acetonitrile containing 0.2% formic acid to maintain a low pH for stable amino acid analysis. Between samples, the column was automatically cleaned with 90% methanol solution followed by 50% isopropanol. The measured spectrometric data were then analyzed with the MetIDQ Software, converted into concentration-values, and provided as pg/mL supernatant.

### 2.5 Statistical analysis

To analyze the proportion of neurons responding to the supernatants from both patient groups and controls, we counted the dye-labeled neurons per ganglion and overlaid the signals with the ganglion image. This allowed us to analyze the response of individual neurons. In the case of spontaneously active neurons, the frequency of action potential discharge following the application of supernatants had to exceed the activity by at least 10% to be counted as a genuine response. In this case, the baseline number of action potentials was subtracted from the number of subsequent supernatant-evoked action potentials. The data are expressed as neuroindex, which describes the product of the mean action potential frequency and the percentage of responding neurons per ganglion (Hz × %). Mast cell density is illustrated as cells/mm^2^ for each intestinal region and patient group. For the determination of histamine content the area under the curve of the peaks in the chromatograms for each supernatant was analyzed and the concentrations were calculated based on the ISTD for histamine and on the weight of the biopsy, and are expressed as pg/mL^*^mg. When comparing three groups, one way analysis of variance or in the case of non-parametric data distribution, Kruskal-Wallis one-way analysis of variance on ranks test was used (SigmaPlot 12.5; Systat Software Inc., San Jose, California). For all tests, a *p*-value < 0.05 was considered significant.

## 3 Results

### 3.1 Nerve activating properties of mucosal biopsy supernatants from IBS-D, MC-D, and non-IBS/non-MC patients

**Figure 1:**
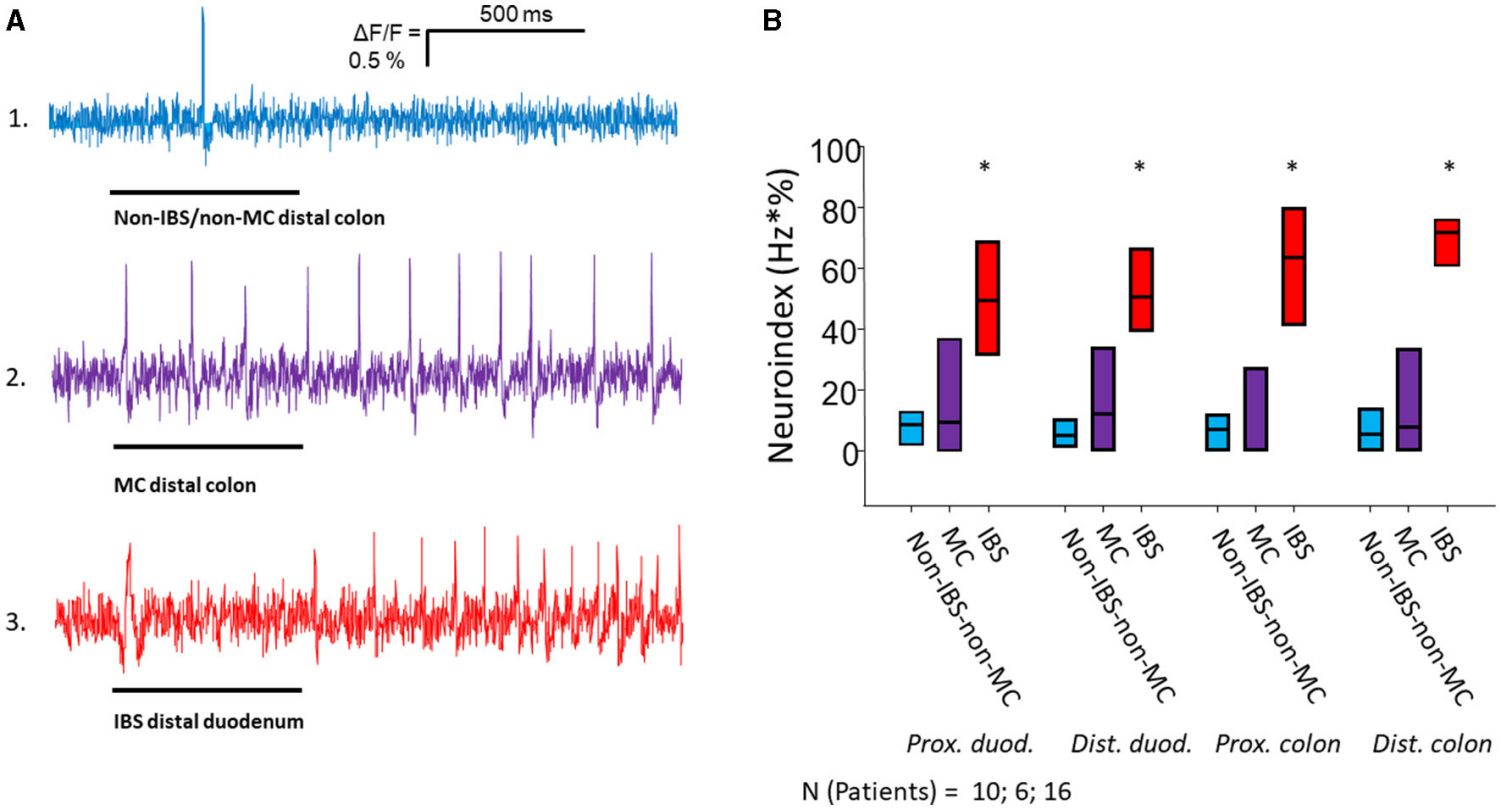
Nerve activating properties of mucosal biopsy supernatants from non-IBS/non-MC, MC-D and IBS-D patients. **(A)** Spike discharge in guinea pig submucous neurons after pressure application of mucosal biopsy supernatants (spritz duration illustrated by black bars below traces) from the distal colon of a non-IBS/non-MC patient (trace 1) and from a MC-D patient (trace 2) in the same neuron. Trace 3 shows spike discharge in another neuron of a separate ganglion after application of mucosal biopsy supernatant from the distal duodenum of an IBS patient. Supernatants from non-IBS-non-MC patients rarely induced neuronal spikes (trace 1). Supernatants from MC patients (trace 2) evoked a spike discharge comparable to supernatants from IBS patients (trace 3), but in a significantly lower percentage of neurons (see Table 2). **(B)** Mucosal biopsy supernatants of IBS-D patients taken from all four locations activate submucous neurons significantly stronger than those of non-IBS/non-MC and MC-D as represented by the neuroindex (* < 0.05).

Application of mucosal biopsy supernatants from IBS-D patients strongly activated submucous plexus neurons, while supernatants from non-IBS/non-MC evoked only marginal responses. The IBS-D-supernatants evoked a remarkably stronger action potential discharge than those from non-IBS/non-MC (*p* < 0.05). This was true for all four intestinal regions. Likewise, supernatants from MC-D patients evoked a higher spike frequency than those from non-IBS/non-MC (*p* < 0.05) in all four regions. However, only a small number of neurons responded to those samples. When analyzing the percentage of responding neurons, it was significantly increased in the case of IBS-D patients compared to both non-IBS/non-MC and MC-D patients (*p* < 0.05), while MC-D patients did not differ from non-IBS/non-MC (*p* > 0.05) (Table 2). A more representative and relevant measure for nerve activating properties, in general, is the neuroindex, which is the product of spike frequency and percentage of responding neurons and may better represent the physiological impact of evoked nerve activity (Figure 1). The neuroindex was significantly higher in response to supernatants from IBS-D patients compared to both supernatants from non-IBS/non-MC or MC-D patients. There was no difference between supernatants from non-IBS/non-MC and MC-D patients (*p* > 0.05).

**Table 2:** Median action potential frequency, percentage of responding neurons, and neuroindex after application of mucosal biopsy supernatants from non-IBS/non-MC, MC-D, and IBS-D.

		**Non-IBS/ non-MC**	**MC-D**	**IBS-D**
Action potential frequency (Hz)	Prox. duod.	0.5 (0.4/0.7)	1.3 (0.8/1.6)^*^	1.5 (1.1/1.9)^*^
	Dist. duod.	0.4 (0.2/0.5)	1.3 (0.6/1.6)^*^	1.1 (1.0/1.4)^*^
	Prox. colon	0.3 (0.2/0.4)	1.3 (1.1/2.5)^*^	1.3 (1.1/1.6)^*^
	Dist. colon	0.3 (0.3/0.4)	1.1 (0.4/1.4)^*^	1.4 (1.2/1.5)^*^
Percentage of responding neurons (%)	Prox. duod.	11 (9/20)^#^	16 (5/29)^#^	50 (40/68)
	Dist. duod.	10 (7/17)^#^	18 (2/36)^#^	58 (43/68)
	Prox. colon	12 (6/17)^#^	9 (6/30)^#^	55 (42/63)
	Dist. colon	11 (9/15)^#^	16 (2/39)^#^	60 (46/64)
Neuroindex (Hz × %)	Prox. duod.	8.7 (2.2/12.6)	9.4 (0/36.8)	49.3 (31.4/68.9)^§^
	Dist. duod.	4.9 (1.2/10.3)	11.9 (0.0/34.1)	50.5 (39.3/66.5)^§^
	Prox. colon	6.9 (0.0/11.9)	0.0 (0.0/27.2)	63.7 (41.3/79.8)^§^
	Dist. colon	5.4 (0.0/14.0)	7.9 (0.0/33.3)	71.9 (60.9/76.0)^§^

^*^p < 0.05 compared to non-IBS/non-MC.

^#^*p* < 0.05 compared to IBS-D.

^§^*p* < 0.001 compared to non-IBS/non-MC and MC-D.

As all MC-D and the majority of IBS-D patients were female, we compared the neuroindex between the three groups in female patients only. The neuroindex (Hz × %) was significantly higher in IBS-D patients compared to both MC-D and non-IBS/non-MC in the duodenum [non-IBS/non-MC: 8.5 (5.1/10.6) vs. MC-D: 9.4 (0.0/23.8) vs. IBS-D: 47.7 (33.4/62.8); *p* < 0.001], proximal colon [non-IBS/non-MC: 6.3 (0.0/13.2) vs. MC-D: 0.0 (0.0/27.2) vs. IBS-D: 64.4 (50.1/88.6); *p* < 0.001[ and distal colon [non-IBS/non-MC: 8.0 (5.1/16.2) vs. MC-D: 7.9 (0.0/33.3) vs. IBS-D: 71.7 (60.5/86.0); *p* < 0.001].

We also analyzed the data without the systemic MC patient. Again, the neuroindex (Hz × %) was significantly higher in IBS-D patients compared to both MC-D and non-IBS/non-MC in the duodenum [non-IBS/non-MC: 7.1 (1.8/10.6) vs. MC-D: 8.0 (0.0/29.1) vs. IBS-D: 49.5 (34.8/66.5); *p* < 0.001], proximal colon [non-IBS/non-MC: 6.9 (0.0/11.9) vs. MC-D: 11.6 (0.0/34.6) vs. IBS-D: 63.7 (41.3/79.8); *p* < 0.001] and distal colon [non-IBS/non-MC: 5.4 (0.0/14.0) vs. MC-D: 7.4 (0.0/39.6) vs. IBS-D: 71.9 (60.9/76.0); *p* < 0.001].

### 3.2 Mast cell count

**Figure 2:**
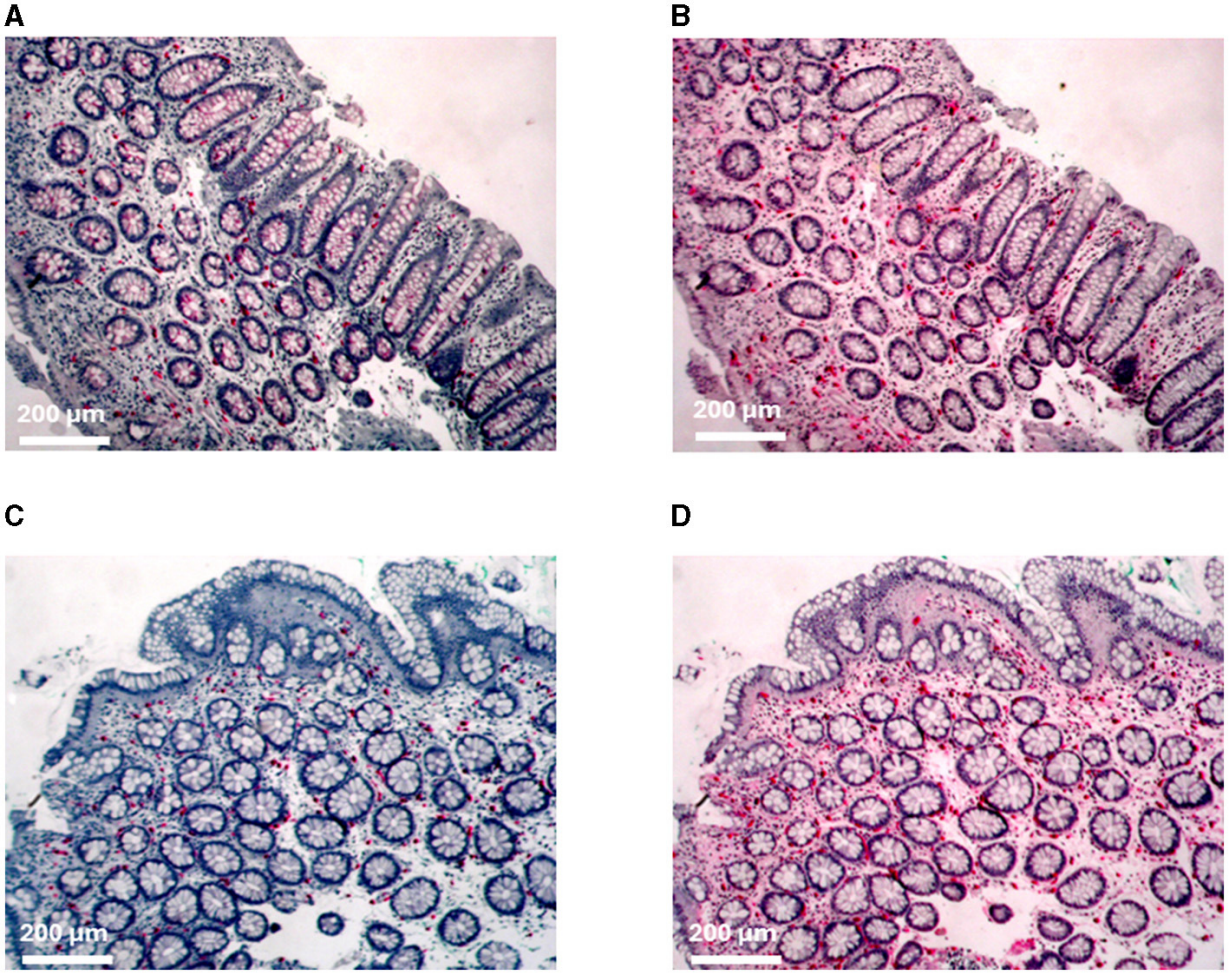
Immunohistochemical staining of mast cells. **(A)** C-kit and **(B)** tryptase staining of the proximal colon from patient MC 1. **(C)** C-kit and **(D)** tryptase staining of the distal colon from patient IBS 11.

Mucosal biopsies from the duodenum, proximal colon (two from each participant), distal colon (two from each participant), and rectum from all patients were immune stained for c-Kit and tryptase (Table 3; Figure 2). The mast cell numbers are presented as mast cells/mm^2^.

**Table 3:** Mast cell numbers counted in the different segments (mean ± SD).

**Mast cells (1/mm^2^)**	**Duodenum**		**Prox. colon**		**Dist. colon**		**Rectum**	
	**c-kit**	**tryp**	**c-kit**	**tryp**	**c-kit**	**tryp**	**c-kit**	**tryp**
Non-IBS/non-MC	369 ± 254	102 ± 106	353 ± 153	117 ± 61	308 ± 134	114 ± 58	231 ± 34	110 ± 54
IBS-D	491 ± 187	90 ± 37	332 ± 146	140 ± 81	317 ± 138	125 ± 93	334 ± 172	208 ± 156
MC-D	325 ± 48	197 ± 96	253 ± 110	170 ± 80	268 ± 109	173 ± 95	305 ± 136	188 ± 93

The numbers of c-Kit- and tryptase (tryp)-positive mucosal mast cells of patients with MC-D and IBS-D, as well as non-IBS/non-MC, showed a strong statistical variance. The mean values are summarized in Table 3. There was no increased or decreased mast cell number in biopsies from the duodenum, proximal colon (c-kit: *p* = 0.456, tryp: *p* = 0.534), distal colon (c-kit: *p* = 0.747, tryp: *p* = 0.551) or rectum (c-kit: *p* = 0.515, *p* = 0.596) of IBS-D and MC-D patients compared to non-IBS/non-MC (duodenum c-kit: *p* > 0.05; tryp: *p* > 0.05; prox. colon: c-kit: *p* = 0.456; tryp: *p* = 0.534; distal colon: c-kit: *p* = 0.747; tryp: *p* = 0.551; rectum: c-kit: *p* = 0.515; *p* = 0.596; respectively).

We calculated the proportion of mast cells based on tryptase and c-Kit labeling of each biopsy, respectively. We could not detect any significant difference between the patient groups in all analyzed biopsies from duodenum (*p* = 0.424), proximal colon (*p* = 0.534), distal colon (*p* = 0.398), and rectum (*p* = 0.777).

### 3.3 Determination of the concentrations of histamine in mucosal biopsy supernatants

Histamine (*p* = 0.562 for proximal duodenum; *p* = 0.082 for distal duodenum; *p* = 0.438 for proximal colon; *p* = 0.375 for distal colon) levels in supernatants from IBS-D and MC-D patients did not differ significantly from each other and from non-IBS/non-MC (Table 4).

**Table 4:** Histamine content of the supernatants from different regions (in median).

	**Histamine (pg/mL** × **mg)**			
	**Proximal duodenum**	**Distal duodenum**	**Proximal colon**	**Distal colon**
Non-IBS/ non-MC	2.3 (1.9/3.1)	2.4 (1.4/5.6)	4.0 (2.4/7.6)	5.9 (2.3/8.6)
MC-D	2.2 (1.0/6.2)	1.6 (0.9/2.5)	5.6 (2.4/12.5)	6.1 (2.6/10.1)
IBS-D	3.0 (2.0/4.0)	3.3 (1.8/4.8)	2.8 (1.9/4.2)	2.9 (1.7/7.5)

## 4 Discussion

In the present study, we analyzed biopsy supernatants from three different patient groups, IBS-D, MC-D, and non-IBS/non-MC, obtained from duodenal and colonic mucosal biopsies. All patients presented GI symptoms, such as diarrhea, abdominal pain, and bloating. Only biopsy supernatants from IBS patients caused significant activation of submucous neurons. Additionally, the nerve-activating effect of duodenal mucosal biopsy supernatants from the same IBS-D patients was comparable to colonic biopsy supernatants. These results demonstrate for the first time that the nerve activating action of IBS-D mucosal biopsy supernatants is a general phenomenon throughout the gut and not restricted to the descending colon. Interestingly, supernatants from MC-D patients markedly activated some of the neurons, but the percentage of responding neurons was dramatically lower than that activated by IBS-D supernatants both in case of the small and large bowel. Consequently, the neuroindex, which is the product of spike frequency and proportion of responding neurons is not significantly elevated by MC-D supernatants and is comparable to non-IBS/non-MC.

As one aim of our study was to collect biopsy supernatants from several small and large intestinal regions, it was not possible to perform biopsy sampling in healthy volunteers. Instead, we used a group of patients who required biopsy sampling because of different gut-related complaints but had neither IBS nor MC. All MC patients suffered from cutaneous mastocytosis except for one with systemic mastocytosis. The significant differences remain when we exclude the patient with systemic mastocytosis. The age of the non-IBS/non-MC patients was comparable to MC but higher than IBS patients, which reflects epidemiology data that mostly younger females suffer from IBS (Enck et al., 2016). Exclusion of the data from male patients allowed two conclusions. Gender is not a confounding variable as the significant differences remain. Moreover, we lost the age differences and can further conclude that age had no effect as the significant differences between the patient groups remain. This agrees with our previous findings that age did not affect the responsiveness of enteric neurons (Breunig et al., 2007; Buhner et al., 2009; Krueger et al., 2016).

As detailed in the introduction, the role of mast cells in IBS pathophysiology and symptoms is still debated (Braak et al., 2012; Sohn et al., 2013). In a group of therapy-resistant IBS patients, 19 out of 20 had symptoms potentially linked to mast cell mediator release, and 11 out of 12 patients had pathologically elevated coagulation and fibrinolysis markers in the blood, which are related to increased mast cell activity (Frieling et al., 2011). However, blood tryptase levels were not elevated. Normal blood tryptase levels do not exclude increased release of mast cell mediators. Mast cells can selectively release their mediators responding to different stimuli (Theoharides et al., 2007), and immature mast cells do not produce tryptase (Qi et al., 2003). The released mediators can be affected by different conditions, which may impede that they reach the blood flow (Molderings et al., 2011). Furthermore, out of the over 60 known mast cell mediators, only a few can be measured with routine commercial techniques (Molderings et al., 2011). We chose to investigate histamine levels and mast cell density in colonic and duodenal mucosal biopsies from all patients in order to check whether or not an increased mast cell number and mediator release are present in these patients. In serial sections we single-labeled mast cells by either c-kit or tryptase. This allowed us to quantify mast cells and to estimate mast cell reactivity by calculation of the c-kit/tryptase ratio. Future experiments should use double labeling to provide direct evidence for altered mast cell reactivity. We have decided to use these two different immunohistological markers to identify mast cells in our tissue samples for the following reasons. First, tryptase is considered a good marker of mast cells, but it is expressed only late in differentiated mast cells (Qi et al., 2003). Furthermore, the staining may be missed if tryptase is already degranulated from the mast cell. C-kit is a receptor of the Kit ligand, which is a major growth factor of human mast cells. Contrary to tryptase, c-kit stains also immature mast cells, but it is relatively unspecific, as it is also expressed in ICC cells of the gut (Chai et al., 2017). It was possible to distinguish the two cell types based on their characteristic morphology. While ICC cells have an elongated, bipolar, or spindle-shaped appearance and are always tryptase negative, mast cells have a clear round appearance. However, only 70–90% of tryptase positive mast cells in the human stomach and colon were stained by c-kit. Therefore the combination of the two methods to refine the number of mast cells seems rational (Qi et al., 2003). We could not detect altered mast cell numbers with any of the two markers, neither in duodenal nor colonic mucosal biopsies from IBS-D and MC-D patients. This leads us to the conclusion that the infiltration of mast cells is not mandatory for the sensitization of enteric neurons. We may exclude that the mast cells were hyperactive. First, the ratio between tryptase and c-kit immune-positive mast cells did not suggest an increased mast cell degranulation, neither in MC-D nor IBS-D patients. Secondly, we could not detect any significantly increased or decreased histamine levels in mucosal biopsy supernatants from any of the patient groups.

In conclusion, mast cells are not involved in nerve activation, at least not in the patient groups enrolled in this study. These results confirm our previous findings that the most decisive components in nerve activation in IBS are proteases, many of which are not released by mast cells but by epithelial cells or other immune cells (Buhner et al., 2018). Furthermore, our data are supported by previous results showing that in combination with tryptase, low concentrations of histamine and serotonin are already sufficient for nerve activation as tryptase potentiates their effect (Ostertag et al., 2017). However, our results cannot explain the observed efficacy of the mast cell stabilizer, ketotifen, in reducing gastrointestinal symptoms in IBS-D patients in a clinical trial (Wang et al., 2020).

In a previous study, we found an association between visceral pain and the degree of enteric and sensory nerve activation by biopsy supernatants (Buhner et al., 2014). Likewise, the number of mast cells in close proximity to mucosal nerve fibers correlate with pain perception in IBS patients (Barbara et al., 2004). Our findings would not suggest a significant role for mast cells or their mediators for the nerve activation evoked by biopsy supernatants. We must admit that it was impossible in our biopsy samples to analyze how close mast cells were to visceral afferent fibers which transmit visceral sensation and pain. Mast cells are mobile, and it may be possible that a closer apposition of mast cells to nerves causes sensitization of nerves, despite the finding that there was no increase in mast cell density, degree of degranulation, or histamine level in our samples.

In our patient groups, diarrhea *per se* was not linked to nerve activation, as mucosal biopsy supernatants from MC-D patients who also had diarrhea did not show the same nerve activating properties. In addition, some non-IBS/non-MC patients had diarrhea, but the supernatants did not evoke a meaningful nerve activation. This is in line with our previous observations that mucosal biopsy supernatants activate enteric neurons significantly stronger than those of healthy controls, independent of bowel habits (Buhner et al., 2009, 2012).

We previously showed that visceral hypersensitivity correlates with the nerve-activating properties of mucosal biopsy supernatants from IBS patients (Buhner et al., 2014). Unfortunately, we do not have any quantitative data on the severity or frequency of abdominal pain or visceral sensitivity in our IBS-D and MC-D patients. Therefore, these parameters cannot be correlated with the nerve activation in the present study.

Our findings highlight IBS as a more extended gastrointestinal disorder rather than a pathology located only in the colon. Our study strongly suggests that excitation of enteric nerves by mediators released from the mucosa occurs throughout the small and large intestines. This novel perspective may help to understand the pathogenesis better and contribute to developing novel therapeutic strategies. In addition, we conclude that GI complaints *per se* are not associated with biopsy supernatant-induced nerve activation, which further stresses the relevance of altered nerve behavior in IBS. We previously reported a comparable nerve activation by biopsy supernatants from IBS and ulcerative colitis patients in remission (Buhner et al., 2018). However, the proteome profile was different and so was the pharmacology behind the nerve activation. This finding, too, stresses the concept that nerve activation by biopsy supernatants possesses features unique to IBS.

## Data availability statement

The raw data supporting the conclusions of this article will be made available by the authors, without undue reservation.

## Ethics statement

The studies involving humans were approved by Ethics Committee of Heinrich-Heine-Universität Düsseldorf under study no. 3166. The studies were conducted in accordance with the local legislation and institutional requirements. The participants provided their written informed consent to participate in this study. The animal study was approved by Bavarian state Ethics Committee (Regierung Oberbayern, which serves as the Institutional Care and Use Committee for the Technical University of Munich). The study was conducted in accordance with the local legislation and institutional requirements under reference number 32-568-2.

## Author contributions

SV: Data curation, Formal analysis, Investigation, Methodology, Writing – original draft. SB: Formal analysis, Investigation, Methodology, Supervision, Writing – review & editing. WG: Formal analysis, Investigation, Methodology, Writing – review & editing. HD: Formal analysis, Investigation, Methodology, Writing – review & editing. TF: Conceptualization, Methodology, Writing – review & editing. MS: Conceptualization, Funding acquisition, Supervision, Writing – original draft, Writing – review & editing. AA: Conceptualization, Funding acquisition, Visualization, Writing – original draft, Writing – review & editing.
